# QuantiFERON-TB Gold Test Conversion Is Associated with Active Tuberculosis Development in Inflammatory Bowel Disease Patients Treated with Biological Agents: An Experience of a Medical Center in Taiwan

**DOI:** 10.1155/2019/7132875

**Published:** 2019-11-03

**Authors:** Hsiang-Chun Lai, Chia-Hsi Chang, Ken-Sheng Cheng, Tsung-Wei Chen, Yuan-Yao Tsai, Jen-Wei Chou

**Affiliations:** ^1^Department of Chinese Medicine, China Medical University Hospital, Taichung, Taiwan; ^2^School of Medicine, China Medical University, Taichung, Taiwan; ^3^Division of Gastroenterology and Hepatology, Department of Internal Medicine, Asia University Hospital, Taichung, Taiwan; ^4^Division of Gastroenterology and Hepatology, Department of Internal Medicine, China Medical University Hospital, Taichung, Taiwan; ^5^Department of Pathology, China Medical University Hospital, Taichung, Taiwan; ^6^Division of Colorectal Surgery, China Medical University Hospital, Taichung, Taiwan; ^7^Taiwan Society of Inflammatory Bowel Disease, Taiwan; ^8^Taiwan Association for the Study of Small Intestinal Diseases, Taiwan

## Abstract

Taiwan has a lower prevalence of inflammatory bowel disease (IBD) and a higher prevalence of tuberculosis (TB) infection than Western countries. The aim of this study was to investigate the prevalence of latent TB (LTB) and active TB infection in IBD patients treated with biological agents. From January 2000 to September 2018, we retrospectively collected data from IBD patients treated with biological agents at a tertiary referral center. Patients underwent a QuantiFERON-TB Gold test (QFT) to screen for TB infection before and after biological treatment courses. The diagnostic age, sex, body mass index, hepatitis B virus infection, biochemistry profile, treatment regimens, and the results of the QFT were analyzed. Overall, 130 IBD patients who received biological treatment were enrolled. The results of the QFT before biological treatment were determined in 120 patients (92%); of these, 10 were positive (8%), 110 were negative (85%), and 10 were indeterminate (9%). Six patients demonstrated seroconversion after biological treatment, as determined by the QFT. Three patients (2.4%) developed active pulmonary TB after biological treatment. In subgroup analysis, the positive QFT patients had a trend of lower baseline serum C-reactive protein and erythrocyte sedimentation rate levels than the negative QFT group. The present study demonstrates that the prevalence of LTB before and after biological treatment is higher in Taiwan than in most Western countries and similar to other Asian countries. Therefore, screening and monitoring of TB infection are necessary for IBD patients before and during biological treatments in Taiwan.

## 1. Introduction

Inflammatory bowel disease (IBD), including Crohn's disease (CD) and ulcerative colitis (UC), is a chronic and relapsing disease with an unknown etiology. Recent advances in biological agents have improved the outcomes of IBD patients, albeit with higher costs [1]. Despite this, biological treatments also carry a risk of life-threatening opportunistic infections, such as new tuberculosis (TB) infection and reactivation of latent tuberculosis (LTB) [2] Prior TB screening and prophylactic treatment for LTB patients are recommended to prevent TB reactivation during biological treatment [3]. Moreover, malnutrition caused by IBD and an immunocompromised state related to hepatitis infection and long-term use of IBD drugs increase the risk of LTB infection [4–6]. A lower prevalence of IBD and a higher prevalence of TB infection and hepatitis B virus (HBV) infection were reported in Asia than in Western countries [7, 8]. Recently, the incidence and prevalence rates of IBD have been reported to be increasing in Taiwan [7]. Thus, adequate surveillance of the TB infection state is crucial for the effective treatment of IBD patients in Taiwan.

Considering previous studies in Taiwan, the majority of the epidemiological studies of IBD were population-based studies retracted from the Taiwan National Health Insurance (NHI) Database. Moreover, biological agents are partially paid for by NHI verification in Taiwan; some IBD patients receive biological treatment by self-paid or private insurance, which might pose a bias in the estimation of TB infection after biological treatments from the Taiwan NHI Database research. This study is aimed at investigating the prevalence of LTB in IBD patients before and after biological treatments and at consequently assessing active TB disease in IBD patients receiving biological treatment at a hospital-based database from the China Medical University Hospital (CMUH).

## 2. Materials and Methods

### 2.1. Study Population

We retrospectively searched the database of chart records from January 2000 through September 2018 in CMUH, a medical center in Taiwan. We identified IBD patients by the International Classification of Disease (2001 version) for disease coding, UC as 556.XX and CD as 555.XX in CMUH chart records. We included patients who were followed up regularly after the original IBD diagnosis or treatment. The diagnostic criteria for IBD was based on a combination of clinical, endoscopic, and pathologic findings and excluded those with an infectious etiology. The follow-up duration of the patients initiated at the time of diagnosis or the first-time visit to our hospital for treatment and ended at the last time recorded in the chart.

We collected clinical data of all IBD patients, including sex, age at diagnosis, age at biological agent usage, body mass index (BMI), alcohol and smoking habits, family history, HBV infection status, biochemistry profile [albumin, C-reactive protein (CRP), erythrocyte sedimentation rate (ESR)], and treatment regimens. The clinical course of all enrolled patients was well recorded and analyzed. Suspicions of TB infection were based on the results of the QuantiFERON-TB Gold test (QFT), which was applied before and after the biological treatment course, and every 6 months thereafter. Indeterminate QFT results were rechecked at least twice. The diagnosis of active TB or LTB disease was confirmed by chest X-ray and serial sputum culture. Patients with positive QFT results were diagnosed with LTB and were treated with anti-TB therapy for 6–9 months before the initiation of treatment with biological agents.

### 2.2. Statistical Methods

With respect to statistical methods, descriptive statistics were presented in the form of mean (standard deviation) and median (interquartile range) for continuous variables, and as frequency and proportion (%) for categorical variables. We compared the characteristics between the two groups by using either a two-sample *t*-test or Wilcoxon rank-sum test for continuous variables, and a chi-square test or Fisher's exact test for categorical variables. All statistical analyses were performed using SAS version 9.4 (SAS Institute Inc., Cary, NC, USA) and R version 3.5.1 (R Foundation for Statistical Computing, Vienna, Austria). The significance level was set at 0.05, and all tests were two-tailed.

### 2.3. Ethics Considerations

This study was approved by the institutional review board of the Research Ethic Committee of China Medical University Hospital in Taiwan (CMUH107-REC1-139).

## 3. Results

Overall, 130 IBD patients who received biological therapy were enrolled in this study. The baseline clinical characteristics of all IBD patients are shown in Table 1. The study included 71 patients with CD and 59 patients with UC, and male patients accounted for most of the study group (75%). The mean diagnostic age of all patients was 37.18 ± 15.96 years, and the mean age at the use of biological agents of all patients was 41.12 ± 15.91 years. The mean follow-up period of all IBD patients was 32.1 ± 24.19 months. The mean ages at biological treatment of CD and UC were 38.18 ± 18.81 years old and 43.44 ± 14.56 years old, respectively. Moreover, the mean follow-up periods of CD and UC were 44.15 ± 25.28 months and 17.59 ± 11.74 months, respectively. The baseline BMI of all IBD patients before starting biological agents was 21.8 ± 4.07 kg/m^2^. In our study, CD patients had a lower baseline BMI compared to the UC group (CD: 21.09 vs. UC: 22.69, *p* = 0.028). In terms of smoking status, the prevalence of current smokers and ex-smokers, and non-smokers was 35% and 65%, respectively. There was no statistically *significant difference* between the CD and UC patients (*p* = 0.381). With regard to alcohol consumption, the prevalence of current drinking and ex-drinking, and no drinking was 85% and 15%, respectively. There was no statistically *significant difference* between the CD and UC patients (*p* = 0.487). Our study showed that 2% of all IBD patients had a family history of IBD, although this was not significantly different between the CD and UC groups (CD: 0% vs. UC: 3%, *p* = 0.204). Furthermore, we reported a 12% rate of HBV infection and 1% rate of historic TB infection. In terms of biochemistry profiles, the CD patients had significantly lower baseline serum albumin levels compared to the UC group (CD: 3.84 ± 0.65 g/dL vs. UC: 4.12 ± 0.66 g/dL, *p* = 0.027). Moreover, CD patients showed higher baseline serum CRP and ESR levels, although this was not significant (*p* = 0.429 and *p* = 0.142, respectively). Patients were prescribed with corticosteroids, 5-aminosalicylic acids, and immunomodulators at a frequency of 82%, 96%, and 56%, respectively. Drug regimens were similar between the two groups, except that CD patients had a significantly higher use of immunomodulators compared to the UC group (CD: 68% vs. UC: 42%, *p* = 0.007). The usage ratio of the biological agents adalimumab, vedolizumab, infliximab, and golimumab was 95%, 8%, 5%, and 2%, respectively.

The QFT results before biological treatment are shown in Table 2. Our study showed that 120 patients (92%) had determinate results: 10 patients (8%) were positive (CD: 6 patients, UC: 4 patients) and 110 patients (85%) were negative (CD: 57 patients, UC: 53 patients). In addition, our study showed that ten patients had indeterminate results (9%) (CD: 8 patients, UC: 2 patients). All the ten patients with positive QFT results had normal chest X-rays and normal serial sputum culture reports. The prevalence of LTB was 8%, and there was no *significant difference* in the QFT results before biological treatment between the CD and UC groups.

The QFT results after biological treatment are shown in Table 3. In total, 123 patients, including 69 CD patients and 54 UC patients, had paired QFT results due to the limitation of follow-up duration. Of all the study participants, 122 patients had determinate results (122/123, 99%): 16 patients (13%) were positive (CD: 11 patients, UC: 5 patients) and 110 patients (86%) were negative (CD: 58 patients, UC: 48 patients). One patient (0.8%) in the UC group had an indeterminate result. Thirteen of the 16 patients with positive QFT results had normal chest X-rays and normal serial sputum culture reports. Three patients (2.4%) demonstrated active TB infection after receiving biological agents (CD: one patient vs. UC: two patients). The detailed information of all patients is summarized in Table 4. The prevalence of LTB was 10.6%, and there was no statistically *significant difference* in QFT results between the CD and UC groups after biological treatment. There was also no significant difference between the active TB infection rate between the CD and UC groups.

A subgroup analysis of the QFT results before biological treatment is shown in Table 5. Patients with positive QFT results before biological treatment had a similar male sex ratio, disease distribution, follow-up period, and BMI compared to the negative group. The positive group had a significantly older diagnostic age (positive: 47.7 ± 15.34 vs. negative: 35.61 ± 15.54, *p* = 0.037) and a significantly older age at biological usage (positive: 53.2 ± 17.08 vs. negative: 39.63 ± 15.34, *p* = 0.035) compared to the negative group. The positive group also had significantly lower baseline serum CRP levels compared to the negative group (positive: 0.89 ± 0.56 vs. negative: 1.98 ± 4.26, *p* = 0.019). There was no statistically *significant difference* in terms of smoking habit, alcohol drinking, and baseline serum albumin and baseline ESR levels between the positive and negative groups.

A subgroup analysis of the QFT results after biological treatment is shown in Table 6. Patients with a positive QFT result after biological treatment had a similar male sex ratio, disease distribution, diagnostic age, age at biological agent use, and BMI compared to the negative group. The positive group had significantly lower baseline serum CRP levels compared to the negative group (positive: 0.68 ± 0.77 mg/dL vs. negative: 2.03 ± 4.30 mg/dL, *p* = 0.006). The positive group also had significantly lower baseline serum ESR levels compared to the negative group (positive: 14.50 ± 8.92 mm/hour vs. negative: 22.28 ± 24.40 mm/hour, *p* = 0.030). However, we found no statistically *significant difference* in smoking habit, alcohol drinking, or baseline serum albumin levels between the positive and negative groups.

## 4. Discussion

The conventional treatment of IBD includes 5-aminosalicylic acids, corticosteroids, and immunomodulators such as azathioprine and methotrexate. However, unsatisfactory control of symptoms and high complication rates in IBD patients remain [9]. In the past two decades, biological agents have provided considerable improvement in the treatment of IBD patients. Despite this, there are some safety concerns about biological treatment, including the possibility of skin lesions, malignancy, infections such as TB, and adverse effects on fertility and pregnancy [10]. In a systematic review conducted by Goletti et al., they concluded non-anti-TNF targeted biologics usage, proper LTBI screening investigations, and adequate preventive TB therapy had the opportunity to reduce the risk of TB reactivation in rheumatic patients requiring biological drugs [11]. Southeast Asia has an epidemic of active TB and LTB infection [8]. Indeed, Zhou et al. reported that the incidence rate of TB infection in Taiwan was 72.5 per 100,000 population in 2005 and 45.7 per 100,000 population in 2015 [12]. Moreover, IBD patients in the prebiologic era appear to be at higher risk of TB infection than the general population [13]. Thus, TB infection is a crucial issue to our daily clinical practice.

In the present study, we found that male patients accounted for most of the IBD patients (75%); this is similar to that reported in a previous study in Asia [14]. Moreover, CD patients had a younger age at biological agents use and had a longer follow-up period. The BMI of all included patients was within a normal range, while CD patients had a lower baseline BMI than the UC group (CD: 21.09 kg/m^2^ vs. UC: 22.69 kg/m^2^, *p* = 0.028). Dong et al. and Yadav et al. also found that CD patients had a lower BMI than UC patients and healthy controls [15, 16]. IBD patients in the current study had elevated baseline serum ESR and CRP levels and a normal baseline albumin level. In the subgroup analysis, CD patients had significantly lower baseline serum albumin levels compared to the UC group (CD: 3.84 ± 0.65 g/dL vs. UC: 4.12 ± 0.66 g/dL, *p* = 0.027); this may be due to the severity of the IBD. Our study demonstrated that immunomodulators were prescribed to CD patients more frequently than to UC patients, which is comparable to the findings of a previous report [17]. As the earliest introduced biological agent in Taiwan, adalimumab was used for the majority of the patients in our study (95%).

The reactivation of LTB and the new occurrence of TB pose a significant risk in IBD patients receiving biological treatment. Indeed, the guidelines of the European Crohn's and Colitis Organization suggest screening of TB prior to antitumor necrosis factor (TNF) therapy, especially in epidemic areas [18]. Latent TB was diagnosed by a combination of history, chest X-ray, tuberculin skin test (TST), and interferon-gamma release assays (IGRA). In Taiwan, the National Immunization Program has provided universal neonatal *Bacillus Calmette*-*Guérin* (BCG) vaccination since 1965 and reached a coverage rate of 99.8% by 2004 [19]. The TST may have applied poor specificity on population of previous BCG vaccine [20]. Thus, several previous studies supported the use of QFT in the population of previous BCG vaccination, and some reports even claimed that a positive QFT result was a better predictor of active TB infection than a positive TST [21, 22]. Lee et al. also demonstrated positive QFT results had better prediction in the development of TB infection in hematopoietic stem cell transplant recipients with 90.7% of BCG vaccination history [23]. Furthermore, the QFT has other advantages over the TST such as the requirement of only one visit, objectivity to get a result by a cut-off value, and a prevention of a cross-reactivation in nontuberculous mycobacteria infection [24].

Since previous reports have indicated that smoking and alcoholism pose a risk to LTB infection [25, 26], we investigated the smoking and alcohol habits of patients in our study. However, we found no *statistically significant difference in the results of the QFT analysis; this may be due to the small study population.* Furthermore, we found that positive QFT patients tended to have lower baseline serum CRP and ESR levels than the negative group. The development of active TB was 2.4% (3/123), and we reported a LTB frequency of 8% before biological treatment and 10.6% after biological treatment. The reports on the epidemiology of TB infection in IBD patients are heterogeneous worldwide. A report from the United States showed that 1.5% of IBD patients had a positive QFT result before using an anti-TNF-*α* agent and a frequency of 0.3% of TB reactivation at the end of the follow-up [27]. Hou et al. reported two active TB patients of 7210 patient years of follow-up on anti-TNF treatment, which was markedly lower than that reported in our study [28]. The positive QFT result in IBD patients after biological treatment was reported as 8.4% in Hungary, which was lower than that in our study [29]. The highest positive rate of the QFT was reported in Turkey by Cekic et al., which demonstrated 44.7% of positive QFT and 4.7% of TB reactivation in IBD patients after anti-TNF-*α* agent treatment [30]. Our findings on the prevalence of LTB before biological treatment were similar to that reported in Korea; Kim et al. reported 5.7% positive QFT and 8% of LTB before biological treatment and 16 active TB infection cases of 801 patient years of follow-up [31]. Moreover, Lee et al. reported only 1.2% of positive QFT and 6% of LTB before biological treatment, and 22.9% of LTB after biological treatment, with a 1.2% rate of active TB disease in China. With overall prophylactic anti-TB treatment and close monitoring, we reported a much lower rate of LTB, but a higher rate of active TB disease after biological treatment than that reported in China. Moreover, male sex is believed to have a higher risk of developing active TB disease from latent status [22]. Thus, higher percentage of active TB disease might be associated with the male predominance in Taiwan's population. In Taiwan, some studies demonstrated a 14.5%-19.6% of positive QFT in immunocompromised groups or high-risk workers [19, 32, 33]. Until now, the prevalence rate of LTB in the general population or IBD patients in Taiwan was unknown. Thus, we provide the first epidemiological report of LTB in IBD patients treated with biological agents in Taiwan.

East Asia has unique epidemiological characteristics in terms of IBD patients, such as male predominance, a more ileal type of CD, higher complication rates, and a higher prevalence of TB disease. The NHI system covers over 99.6% of the Taiwanese population and partially pays for biological agents; this means that our study population received biological agents without delay and had a higher biological agent usage compared to other countries. However, some patients use self-paid biological agents, which might pose a bias in the estimation of the epidemiological data on TB infection. Moreover, our study establishes a longitudinal follow-up of IBD patients who received biological agents and also provides laboratory data analysis. We found that QFT-positive IBD patients tended to have lower baseline serum CRP and ESR levels compared to the negative group. We hypothesized that this effect may have been influenced by the balance of T helper cells 1 (Th1) to T helper cells 2 (Th2). The QFT detects interferon-gamma, the main cytokine involved in the immune response against TB infection and regulated Th1 cell differentiation [34, 35]. On the other hand, CRP was found to suppress Th1 cell differentiation [36]. Thus, we hypothesize that lower baseline serum CRP levels prompt Th1 pathways in IBD patients leading to positive QFT results. However, the exact mechanism remains unclear and requires further studies with a larger population, as well as laboratory work in order to delineate the underlying mechanisms.

Nevertheless, our study has several limitations. First, although our hospital is the biggest tertiary hospital in central Taiwan, this hospital-based study had a relatively small sample size and was retrospective in nature. Thus, more prospective population-based studies at the national level are required in order to identify the epidemiological report of TB infection in IBD patients in Taiwan. Second, the mean follow-up period was 32.1 months; this was a restricted time in terms of the assessment of TB infection in IBD patients who received many courses of biological agents. Therefore, we cannot estimate the incidences in our study group because of the short follow-up period, and further investigation is needed. Third, a positive QFT result was defined by cut-off values in our data analysis, and there is a potential of bias and misclassification caused by limited false positive and false negative rates. Thus, both Nemes et al. and Chiacchio et al. had mentioned the gray zone of QFT assay [37, 38]. Chiacchio et al. even concluded that patients with immune-mediated inflammatory diseases were more likely to fall in the gray zone of QFT assay compared to the controls [38]. The uncertain zone in our present study was grouped in the “indeterminate” group. However, we did not collect the exact quantitative data in our present study. Until now, there is no gold standard testing for LTB infection or more suitable and convenient tools than QFT in a routinely BCG-vaccinated area. The diagnosis of LTB was based on the combination of positive QFT, normal finding of chest X-ray, and normal serial sputum culture. Moreover, we would provide additional LTB evaluation according to patient's self-report. Further studies with more advanced diagnostic tools are needed in order to more accurately identify LTB infection in IBD patients.

## 5. Conclusions

In summary, our study demonstrated that the prevalence of LTB in Taiwan, both before and after treatments, is higher than in most Western countries and similar to other Asian countries. In this study, active TB infection was found to be higher than that suggested by previous reports after receiving biological treatment due to the male predominance of our population. Moreover, positive QFT patients in our study tended to have lower baseline serum CRP and ESR levels than the negative group. We suggest that this effect is in part due to the balance of Th1/Th2 cells, although further studies are required in order to fully validate this hypothesis. Screening and monitoring of TB infection are necessary for IBD patients both at the start and during biological treatments in Taiwan. To the best of our knowledge, this is the first hospital-based, retrospective study assessing the prevalence of LTB and active TB in IBD patients receiving biological treatment and the first QFT epidemiological report in Taiwan.

## Figures and Tables

**Table 1:** Clinical characteristics of patients with inflammatory bowel disease.

Characteristics	Total IBD (*n* = 130)	CD (*n* = 71)	UC (*n* = 59)	*p* value (CD vs. UC)
Male gender, *n* (%)	97 (75)	54 (76)	43 (73)	0.832
Diagnostic mean age (years) (SD)	37.18 (15.96)	36.18 (17.46)	38.37 (14.01)	0.429
Mean age at biological agent (years) (SD)	41.12 (15.91)	39.18 (16.81)	43.44 (14.56)	0.124
Mean follow-up period (months) (SD)	32.1 (24.19)	44.15 (25.28)	17.59 (11.74)	**<0.001**
BMI (kg/m^2^), mean (SD)	21.8 (4.07)	21.09 (3.92)	22.69 (4.11)	**0.028**
Smoking				
No smoking, *n* (%)	84 (65)	43 (61)	41 (69)	0.381
Ex-smoking and current smoking, *n* (%)	46 (35)	28 (39)	18 (31)	
Alcohol				
No drinking, *n* (%)	110 (85)	62 (87)	48 (81)	0.487
Ex-drinking and current drinking, *n* (%)	20 (15)	9 (13)	11 (19)	
Family history of IBD, *n* (%)	2 (2)	0 (0)	2 (3)	0.204
Albumin (g/dL), mean (SD)	3.96 (0.66)	3.84 (0.65)	4.12 (0.66)	**0.027**
CRP (mg/dL), mean (SD)	1.9 (3.99)	2.18 (3.95)	1.6 (4.05)	0.429
ESR (mm/hour), mean (SD)	21.65 (23.06)	24.91 (26.38)	18.51 (19.04)	0.142
Comorbidity				
HBV carrier, *n* (%)	15 (12)	8 (11)	7 (12)	1.000
Old TB	1 (1)	1 (1)	0 (0)	1.000
Conventional medicines				
Steroids, *n* (%)	107 (82)	57 (80)	50 (85)	0.665
5-ASAs, *n* (%)	125 (96)	66 (93)	59 (100)	0.063
Immunomodulators, *n* (%)	73 (56)	48 (68)	25 (42)	**0.007**
Biological agent selection				
Adalimumab, *n* (%)	124 (95)	69 (97)	55 (93)	0.410
Infliximab, *n* (%)	7 (5)	6 (8)	1 (2)	0.126
Vedolizumab, *n* (%)	11 (8)	6 (8)	5 (8)	1.000
Golimumab, *n* (%)	2 (2)	0 (0)	2 (3)	0.204

5-ASAs: 5-aminosalicylic acids; BMI: body mass index; CD: Crohn's disease; CRP: C-reactive protein; ESR: erythrocyte sedimentation rate; HBV: hepatitis B virus; IBD: inflammatory bowel disease; TB: tuberculosis; UC: ulcerative colitis.

**Table 2:** Results of QuantiFERON-TB Gold test before biological treatment.

Characteristics	Total IBD (*n* = 130)	CD (*n* = 71)	UC (*n* = 59)	*p* value (CD vs. UC)
Determinate	120 (92)	63 (89)	57 (97)	0.111
Positivity, *n* (%)	10 (8)	6 (8)	4 (7)	1.000
Negativity, *n* (%)	110 (85)	57 (80)	53 (90)	0.208
Indeterminate, *n* (%)	10 (8)	8 (11)	2 (3)	0.111

CD: Crohn's disease; IBD: inflammatory bowel disease; TB: tuberculosis; UC: ulcerative colitis.

**Table 3:** Results of QuantiFERON-TB Gold test after biological treatment.

Characteristics	Total IBD (*n* = 123)	CD (*n* = 69)	UC (*n* = 54)	*p* value (CD vs. UC)
Determinate	122 (99)	69 (100)	53 (97)	0.140
Positivity, *n* (%)	16 (13)	11 (16)	5 (9)	0.288
Negativity, *n* (%)	110 (86)	58 (84)	48 (89)	1.000
Indeterminate, *n* (%)	1 (0.8)	0 (0)	1 (2)	0.454
Latent TB	13 (10.6)	10 (14.5)	3 (5.6)	0.141
Active TB disease	3 (2.4)	1 (1.4)	2 (3.7)	0.590

CD: Crohn's disease; IBD: inflammatory bowel disease; TB: tuberculosis; UC: ulcerative colitis.

**Table 4:** Clinical characteristics of patients with active tuberculosis infection after biological treatment.

Case number	#8	#21	#53
Disease type	UC	UC	CD
Sex	Male	Male	Male
Diagnostic age (years)	47	37	24
Age at biological agent (years)	51	39	24
Diagnosis interval of TB (months)	6	24	21
BMI (kg/m^2^)	24.1	20.1	24.1
Comorbidity	HBV	No	No
Concomitant treatment	Mesalazine, azathioprine	Mesalazine, azathioprine, prednisolone	Mesalazine, mercaptopurine
Biological agent ∗ period (months, m)	Adalimumab ∗ 6 m	Adalimumab ∗ 6 m Adalimumab ∗ 4 m Infliximab ∗ 10 m	Adalimumab ∗ 12 m Adalimumab ∗ 5 m
TB exposure	No	No	No
TB history	No	No	No
BCG vaccination	Yes	Yes	Yes
Pre-QuantiFERON-TB test	Negative	Negative	Negative
Post-QuantiFERON-TB test	Positive	Positive	Positive
Anti-TB prophylaxis	No	No	No
TB location	Pulmonary	Pulmonary	Pulmonary
Diagnostic method of TB infection	CXR, chest CT, sputum AFS, sputum TB PCR, sputum culture	CXR, sputum AFS, sputum TB PCR, sputum culture	Sputum culture
Symptoms	Fever	Fever, dyspnea	No
Course of TB treatment	Cured	Cured	Cured

AFS: acid-fast stain; BCG: Bacillus Calmette-Guérin; BMI: body mass index; CD: Crohn's disease; CT: computed tomography; CXR: chest X-ray; HBV: hepatitis B virus; PCR: polymerase chain reaction; TB: tuberculosis; UC: ulcerative colitis.

**Table 5:** Subgroup analysis of QuantiFERON-TB Gold test results before biological treatment in IBD patients.

Characteristics	Positivity (*n* = 10)	Negativity (*n* = 110)	*p* value
Male sex, *n* (%)	9 (90)	81 (74)	0.448
Diagnosis			
CD	6 (8)	57 (80)	0.746
UC	4 (7)	53 (90)	0.746
Diagnostic mean age (years) (SD)	47.7 (15.34)	35.61 (15.54)	**0.037**
Mean age at biological agent (years) (SD)	53.2 (17.08)	39.63 (15.34)	**0.035**
BMI (kg/m^2^), mean (SD)	21.58 (2.77)	22.05 (4.21)	0.637
Smoking			
No smoking, *n* (%)	4 (40)	71 (65)	0.173
Ex-smoking and current smoking, *n* (%)	6 (60)	39 (35)	
Alcohol			
No drinking, *n* (%)	7 (70)	94 (85)	0.195
Ex-drinking and current drinking, *n* (%)	3 (30)	16 (15)	
Albumin (g/dL), mean (SD)	3.96 (0.54)	4.03 (0.64)	0.702
CRP (mg/dL), mean (SD)	0.89 (0.56)	1.98 (4.26)	**0.019**
ESR (mm/hour), mean (SD)	22.56 (14.87)	19.4 (21.82)	0.573

BMI: body mass index; CD: Crohn's disease; CRP: C-reactive protein; ESR: erythrocyte sedimentation rate; IBD: inflammatory bowel disease; TB: tuberculosis; UC: ulcerative colitis.

**Table 6:** Subgroup analysis of QuantiFERON-TB Gold test results after biological treatment in IBD patients.

Characteristics	Positivity (*n* = 16)	Negativity (*n* = 106)	*p* value
Male, *n* (%)	14 (88)	77 (73)	0.354
Diagnosis, *n* (%)			
CD	11 (16)	58 (84)	0.418
UC	5 (9)	48 (89)	0.418
Diagnostic mean age (years) (SD)	43.63 (15.8)	35.98 (16.10)	0.087
Mean age at biological agent (years) (SD)	47.88 (15.85)	39.62 (15.56)	0.066
BMI (kg/m^2^), mean (SD)	21.29 (3.12)	22.07 (4.20)	0.385
Smoking			
No smoking, *n* (%)	8 (50)	71 (67)	0.296
Ex-smoking and current smoking, *n* (%)	8 (50)	35 (33)	
Alcohol			
No drinking, *n* (%)	12 (75)	90 (85)	0.297
Ex-drinking and current drinking, *n* (%)	4 (25)	16 (15)	
Albumin (g/dL), mean (SD)	3.95 (0.55)	3.95 (0.70)	0.964
CRP (mg/dL), mean (SD)	0.68 (0.77)	2.03 (4.30)	**0.006**
ESR (mm/hour), mean (SD)	14.50 (8.92)	22.28 (24.40)	**0.030**

BMI: body mass index; CD: Crohn's disease; CRP: C-reactive protein; ESR: erythrocyte sedimentation rate; IBD: inflammatory bowel disease; TB: tuberculosis; UC: ulcerative colitis.

## Data Availability

The clinical data used to support the findings of this study are restricted by the institutional review board of the research ethics committee of China Medical University Hospital in order to protect the patients' privacy. Data are available from the corresponding author (Dr. Jen-Wei Chou, codecol@yahoo.com.tw) for researchers who meet the criteria for access to confidential data.
